# Evolutionary considerations on the origin of peroxisomes from the endoplasmic reticulum, and their relationships with mitochondria

**DOI:** 10.1007/s00018-014-1640-1

**Published:** 2014-05-17

**Authors:** Toni Gabaldón

**Affiliations:** 1 Bioinformatics and Genomics Programme, Centre for Genomic Regulation (CRG), Dr. Aiguader, 88, 08003 Barcelona, Spain; 2 Universitat Pompeu Fabra (UPF), 08003 Barcelona, Spain; 3 Institució Catalana de Recerca i Estudis Avançats (ICREA), Pg. Lluís Companys 23, 08010 Barcelona, Spain

**Fig. 1:**
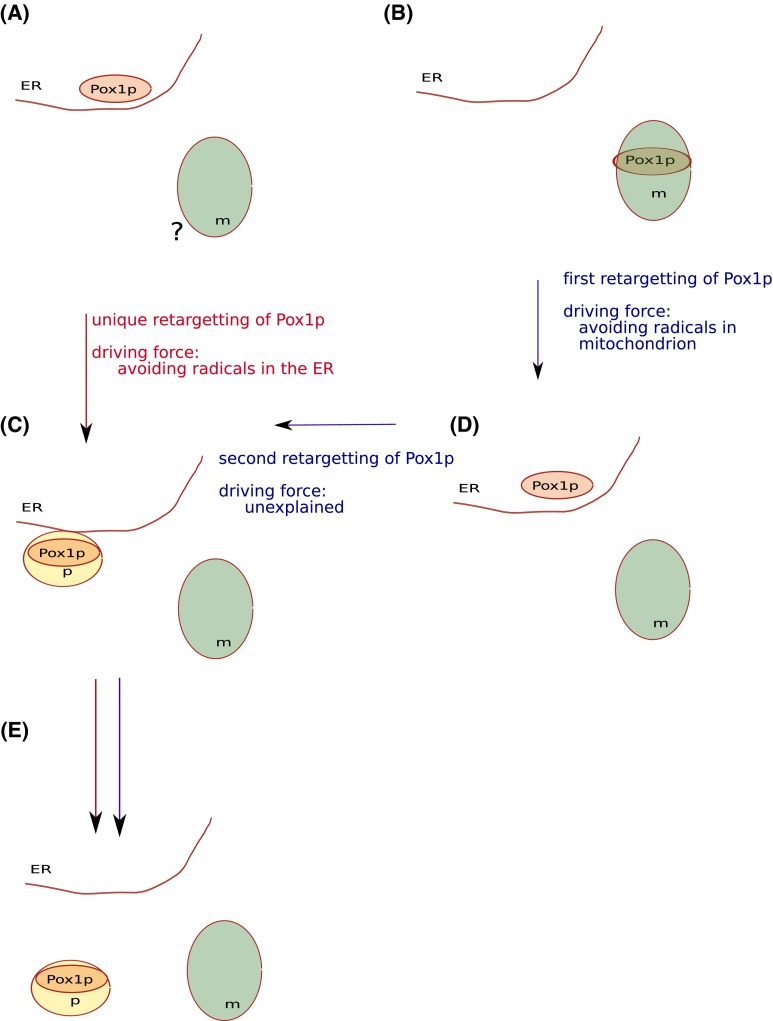
Schematic representation of the two alternative models explaining the origin of peroxisomes. Putative steps are represented from top (past) to bottom (present). Endoplasmic Reticulum (*ER*), Mitochondrion (*m*), and Peroxisomal (*p*) organelles are represented, as well as the corresponding location of Pox1p. The two scenarios share some steps and are indicated by *red* (Gabaldón’s) or *blue* (Speijer’s) *arrows*. Retargeting(*s*) of Pox1p and supposed driving forces are indicated in the corresponding steps, next to the *arrows*. **a** Initial stage in Gabaldon’s model. Pox1p has an ancestral endomembrane location, no assumption is made on whether the mitochondrion was present at that stage (question mark); **b** initial stage in Speijer’s model, Pox1p has an ancestral mitochondrial location. **c** Origin of the peroxisome from the endomembrane system; **d** an intermediate step involving the retargeting of Pox1p to the endomembrane system, necessary only in Speijer’s model. **e** a Pox1p-bearing peroxisome may be the recipient of additional enzymes from the mitochondrion and elsewhere

I recently wrote a perspective paper with the intention of contributing to the debate on the origin of peroxisomes [1]. A subsequent comment by Dr. Speijer [2], suggested that his previous work [3] was not sufficiently discussed. I must apologize for this, as given the space constraints I could not dedicate enough space to all previous work. I take this opportunity to further discuss the issue of the metabolic connection between mitochondria and peroxisomes in relation to the origin of the latter. In his paper, Dr. Speijer observes that the retargeting of part of the beta-oxidation pathway from the mitochondrion to the peroxisome is advantageous to the cell, and proposes that this would explain the origin of the peroxisome. However, no further details on how this may have occurred are provided. In my opinion, the proposed selective force serves to explain why part of the beta-oxidation of fatty acids moved out of the mitochondrion, but not why or how peroxisomes were formed from the endomembranous system, which is the question at hand. In addition, when we contrast with existing data the idea that the retargetting of part of the beta-oxidation from the mitochondrion directly created the peroxisomes, we encounter some important problems, which I outline below. Two possible alternative models are considered that account for the origin of peroxisomes and involve a different number of steps (Fig. 1, red and blue paths), this model would correspond to the scenarios assumed by my proposed model [1] and that of Dr. Speijer [2].

If an origin of peroxisomes from the endomembrane system is assumed [4], one would need to invoke two steps (Fig 1, blue path): first, the relevant beta-oxidation enzymes—at least Pox1p—would have moved to the endoplasmic reticulum to then originate the peroxisomes. This scenario leaves us with the problem of explaining how peroxisomes would have originated from the endomembranous system, because the proposed driving force—to avoid radical formation in the mitochondrion—would have disappeared in the first step. This is the reason why I had previously considered that this model did not actually explain the origin of peroxisomes from the endomembranous system. Secondly, if radical formation is also problematic in the endoplasmic reticulum and this acted as a driving force to separate peroxisomes from the endoplasmic reticulum—as I indeed propose for the first time in my model [1]—, then, in a two-step scenario it would be difficult to explain the selective advantage of this intermediate state. The principle of parsimony, or Occam’s razor, is a general guiding principle in science and, in the absence of additional data supporting such two-step scenario, one should favor simpler models. The fact that the retargeting of pathways has indeed occurred many times does not imply that we should freely consider as many retargeting events as we need to explain our model of choice. In evolution, each intermediate step should have had to survive for some time, and we expect each of the steps to be advantageous over the previous one, or at least not be deleterious.

**Fig. 2:**
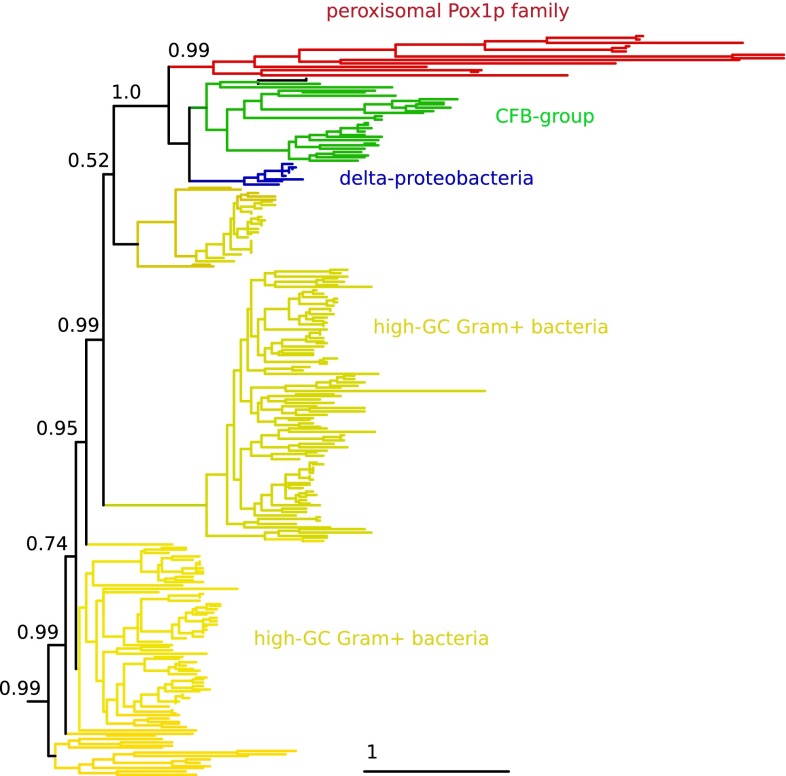
Phylogenetic tree of eukaryotic Pox1p family members and their closest 400 prokaryotic trees showing that Pox1p ancestry cannot be traced back to alpha-proteobacteria. The tree was build as follows. *Saccharomyces cerevisiae* Pox1p sequence was used in a blast search against NCBI nr database. Hits in *Saccharomyces cerevisiae*, *Kluyveromyces lactis, Arabidopsis lyrata,* and *Pongo abelii*–eukaryotic species were chosen to minimize the presence of isoforms—as well as the 400 first, top-scoring prokaryotic hits were selected for further analysis. Selected sequences were aligned with MUSCLE [10], and phylogenetic reconstruction was performed with PhyML [11] using the LG model with four rate categories and estimating the alpha parameter and the proportion of invariable sites from the data. Only the seven closest partitions in which eukaryotes are nested are shown. The full tree in Newick format is included in supplemental. Support in the relevant branches is shown using aLRT values (one is maximal support), and the clades are colored according to the group that is most represented (>90 % of the sequences in the clade)

But perhaps the hardest evidence arguing against the direct origin of peroxisomes from the retargeting of part of mitochondrial beta-oxidation is of another nature. The key enzyme implied by both Speijer’s model and my own model is the one catalyzing the first step of the peroxisomal beta-oxidation pathway: the H_2_O_2_-producing acyl-CoA oxidase (Pox1p in yeast). If this enzyme had originated from diversification by duplication and retargeting of the equivalent enzymes in the mitochodrion, as implied by Speijer’s model, one should expect this to be reflected by its phylogenetic signal. However, a simple blast search reveals that peroxisomal acyl-CoA have their closest homologs among bacteria (e-value 2 × 10^−63^ for the closest bacterial homolog), while hits to the mitochondrial homologs are much less significant (1 × 10^−8^ for the closest human mitochondrial acyl-CoA). This shows that acyl-CoA oxidases in mitochondrion and peroxisomes have a distinct phylogenetic origin. Furthermore, if Pox1p was carried by the mitochondrial ancestor one would expect it to show alpha-proteobacterial ancestry. However, as noted earlier [1, 5–7], this enzyme does not show any phylogenetic proximity to alpha-proteobacteria—where it has few distant homologs the most similar of which with an e-value of 10^−13^. Rather, the closest bacterial homologs of this enzyme belong to CFB group bacteria, delta-proteobacteria, and high-GC Gram+ bacteria (Fig. 2). This implies that this enzyme has radically different phylogenetic origins as compared to its mitochondrial counterparts and that there is no support for the idea that its original location was mitochondrial.

Note that my opposition to the idea that peroxisomes directly resulted from the retargeting of part of the beta-oxidation pathway from the mitochondrion is perfectly compatible with the realization that many enzymes did move from the mitochondrion to the peroxisome, an idea that my earlier work helped to put forward [6, 8]. Indeed, there is solid evidence that multiple enzymes have relocated from the mitochondrion to the peroxisome at various lineages and evolutionary periods [4, 6]. There is truly a very important metabolic and evolutionary inter-play between peroxisomes and the mitochondrion, but this is compatible with many alternative scenarios for the origin of the peroxisome. Remarkably, retargeting of enzymes of alpha-proteobacterial origin into the peroxisome is ancient and involves one enzyme of the beta-oxidation pathway (Pox2p). However, this is not the enzyme generating oxygen radicals—the driving force assumed in both models. In addition, the enzyme that does produce the damaging radicals, Pox1p, is involved in other pathways besides degradation of fatty acids through beta-oxidation, for instance in the synthesis of poly-unsaturated fatty acids [9]. Given its very distinct phylogenetic origin, it is reasonable to think that Pox1p was not involved in the degradation of fatty acids through beta-oxidation until the key enzymes, such as Pox2p, were retargeted from the mitochondrion. A plausible scenario, therefore, is that Pox2p moved to an already existing peroxisome, initially originated to separate the production of oxygen radicals from the endoplasmic reticulum. The relocation of the multifunctional enzyme Pox2p from the mitochondrion, as supported by an alpha-proteobacterial origin, into an already formed peroxisome would have enabled the efficient oxidation of very long fatty acids outside the mitochondrion by coupling to the already existing peroxisomal activity of Pox1p. This scenario is simpler in that it does involve only one retargeting step of a single mitochondrial enzyme, and should be preferred in the absence of additional data pointing to an ancestral mitochondrial location of Pox1p. In addition, it does not couple the origin of the peroxisomes with that of the mitochondrion, and thus it is free from assumptions of which organelle came first.
